# Increased Milk Yield and Reduced Enteric Methane Concentration on a Commercial Dairy Farm Associated with Dietary Inclusion of Sugarcane Extract (*Saccharum officinarum*)

**DOI:** 10.3390/ani13203300

**Published:** 2023-10-23

**Authors:** Awais Ahmed, Matthew Flavel, Shane Mitchell, Gregor Macnab, Manisha Dunuarachchi Dunuarachchige, Aniruddha Desai, Markandeya Jois

**Affiliations:** 1 Department of Microbiology, Anatomy, Physiology and Pharmacology, La Trobe University, Bundoora, VIC 3086, Australia; 17558387@students.latrobe.edu.au (A.A.); m.jois@latrobe.edu.au (M.J.); 2 The Product Makers Bioactive Division, The Product Makers Pty Ltd., Melbourne, VIC 3173, Australia; gmacnab@tpm.com.au; 3 Poly Gain Pte. Ltd., Singapore 138538, Singapore; smitchell@polygain.com; 4 Centre for Technology Infusion, La Trobe University, Bundoora, VIC 3086, Australia; m.dunuarachchige@latrobe.edu.au (M.D.D.); a.desai@latrobe.edu.au (A.D.)

**Keywords:** methane, milk yield, polyphenols, sugar cane, mastitis

## Abstract

**Simple Summary:**

Enteric methane emissions from ruminants have emerged as a major challenge to the global agriculture industry. However, the lack of tools available to commercial farmers to measure and mitigate these emissions is preventing this challenge from being addressed. This study aimed to evaluate natural sugarcane extract’s potential to mitigate these emissions in a commercial dairy environment and assess any impact on milk production and composition. The results of this study indicate a significant increase in milk production, with less methane detected across the herd. Bulk tank somatic cell counts were also reduced indicating improved udder health of cows.

**Abstract:**

(1) Background: The purpose of this study was to assess the influence of a natural sugarcane extract (Polygain™) on milk production, milk composition and methane emissions on a commercial dairy farm. (2) Methods: A three-week baseline was established for lactating Holstein × Friesian animals. Following this baseline period, these animals were fed Polygain™ at 0.25% of their estimated dry matter intake for 3 weeks. Methane concentration in the feed bin was determined at each milking using the Gascard NG Infrared Sensor (Edinburgh Sensors LTD). (3) Results: During the intervention phase milk yield increased significantly from 26.43 kg to 28.54 kg per cow per day, whilst methane emissions and bulk tank somatic cell counts decreased significantly in the intervention phase. For methane concentration, an average of 246 ppm during the baseline periods reduced to an average of 161.09 ppm during the intervention phase. For the bulk tank somatic cell counts, the average was observed at 283,200 during the baseline and reduced to an average value of 151,100 during the intervention phase. (4) Conclusions: The natural sugarcane extract was shown to have the potential to mitigate enteric methane emissions while also increasing production and animal wellbeing outcomes in a commercial dairy setting.

## 1. Introduction

It has been estimated that the total global production of red meat and dairy will need to increase by 76% and 63%, respectively in order to meet the global demand for food by 2050 [1,2]. However, the current contribution of enteric fermentation and manure management contributes 1/3 of anthropogenic methane emissions [3].

Therefore, a new strategy to increase ruminant production whilst decreasing the environmental impact is required.

There have been a variety of strategies suggested in order to mitigate enteric methane from ruminants. This has included feed supplements [4,5,6], genetics [7,8] vaccination and grazing strategies [9]. To date, these strategies have not wholly addressed the global enteric methane challenge, whilst maintaining or increasing production output. Therefore, new approaches that have the capability to be effective under commercial farm conditions are actively being sought out. 

Dairy remains a vital contributor to global food production and therefore any new approach to address the environmental challenges must maintain or ideally improve the economic and social viability of this industry. This viability must be demonstrated at multiple different stages across the food supply chain and the different requirements at each stage should be considered. Effects on profitability and animal health and welfare would be critical considerations at the farm level for a new solution to the challenge of enteric methane emissions. Consumer demand for environmentally responsible products can help assist in enhancing the dairy industries profitability [10]. Mitigating enteric methane emissions is one aspect of meeting the demands of these consumers. However, complementary issues such as usage of antibiotics and synthetic compounds, and the sustainability of feed ingredients used for this purpose have also been identified as valuable to these consumers [11,12]. Therefore, a solution that is natural, non-antibiotic and can be sourced sustainably at scale from an underutilized raw material that is produced by a pre-existing agricultural industry could potentially meet these consumer demands if determined to be efficacious and cost effective. 

There is a growing body of evidence that dietary polyphenols have the potential to have an impact on the production efficiency, health and wellbeing of production animals. Polygain™ is a natural extract from sugarcane enriched with polyphenols and its effects have been studied across a range of production animal species [13,14,15,16]. These previous studies have indicated a significant and beneficial effect on feed conversion efficiency, improved growth rate, animal health and welfare outcomes. Beyond the effect on production efficiency, the question has been raised whether plant extracts that will contain a vast profile of polyphenols may have the ability to alter rumen fermentation and lower methane production in the rumen [17]. Recently, it was reported that Polygain™, polyphenol rich sugar cane extract was observed to have a significant effect on methane emissions and production outcomes in ruminants under controlled experimental conditions [18]. This previous study determined that 0.25% of total dry matter intake was a commercially viable and effective amount to be included in the diet of ruminants. Therefore, the aim of this study was to assess whether any of the benefits to production outcomes or methane mitigation could be translated to a commercial dairy farm environment. Our hypothesis was that Polygain™ would (1) reduce enteric methane, (2) would not have any negative impact on animal production, and (3) would not have a negative impact on udder health. 

## 2. Materials and Methods

### 2.1. Trial Site and Design

The study took place on a commercial dairy farm located southeast of Victoria, Australia. Because this trial was conducted in a commercially operating dairy farm, cows were added to the herd throughout the trial period and were also moved to adjoining properties throughout the trial as part of normal management practices. Animals that required changes to management practices throughout the trial, such as changes to diet or relocation to other properties, were excluded from analysis. In total, 31 cows were observed to stay on the farm for the entire trial period without changes to management practices. The data collected from these cows were therefore suitable to be used for analysis. These animals were able to access the milking barn at all times of the day to be milked. A total of 4 automatic milking systems (LELY) were available to be used at all times throughout the trial and would be fed an allocation of their concentrate ration at this time. The animals visited the milking robots an average of 2.3 times per day throughout the baseline and trial phase of the study. There was no significant difference in the number of times the animals attended the milking parlor throughout the trial. All cows included in the trial were Holstein-Friesians selected from early, mid and late lactation stages (average days in lactation, 240 days, average number of lactations, 3.58). All of the cows were provided with partial mixed ration (PMR) ad libitum and each cow was allocated a concentrate ration based on milk production. The concentrate was supplied by a commercial animal feed company (Browns Stockfeed). The composition of the concentrate is as follows: crude protein 12.79%, starch 60.94%, metabolizable energy 12.92 mj/Kg, calcium 1.81%, phosphorous, 0.34%, magnesium 0.47%, dry matter 88.55%, neutral detergent fibre 10.17%, rumen undegraded protein 26.46%, fat 2.33%, Cu 5.83 mg/kg, magnesium 36.29 mg/kg, selenium 0.14 mg/kg, zinc 25.90 mg/kg. The concentrate was provided to the animals during milking time in the feed bin and this was also the location of the methane sampling and measurement. Three weeks of baseline measurements were recorded, followed by a 3-week intervention period of the sugarcane extract (Polygain™). During the baseline period, the daily average temperature ranged from a minimum of 10 °C to a maximum 16.7 °C and 3.4 mm of rainfall. This is compared to the climactic conditions in the intervention phase with a daily average temperature ranging between 13.05 °C and a maximum of 15.05 °C and 3.7 mm of rainfall. 

### 2.2. Polygain Feeding

The sugarcane extract (Polygain™) was included in the animal’s diet at 0.25% of total estimated dry matter intake (DMI). This was based on previous work in earlier ruminant studies [18]. Based on an estimation of the average combined intake of pasture and concentrate of 20 kg per animal, 50 g of sugarcane extract (Polygain™) was required to be delivered via the concentrate. Animals were on average consuming 5 kg of concentrate per day and therefore the sugarcane extract (Polygain™) was added at 10 g/kg of feed for the dietary intervention phase of the trial. Polygain™ is a dark brown liquid that consists of a complex mixture of natural phytochemicals.

### 2.3. Methane Data Collection

Methane concentration in feed bins during milking was determined by the “Sniffer method” as described in [19,20,21]. Briefly, an infrared methane analyzer (Guardian Plus; Edinburgh Instruments Ltd., Livingstone, UK) was outside the feed bin of each milking robot with a sampling tube extended into the feed bin. The methane analyzer had a measurement range of 0–1% or 0–10,000 ppm. A 3 m length of polyethylene tube with an 8 mm diameter was connected to the inlet port of the gas analyzer. Air was continuously sampled from the feed bins of robots during the trial at a flow rate of 1 L/min. The sampling point was positioned approximately l5cm from the rim of the feed bin, opposite to the side of feed dispensing. A 50 mm inline filter was installed in the tube between the air sampling point in the feed bin and second filter inside the gas analyzer inlet to avoid feed particles, dust and moisture from entering the gas analyzer and causing measurement interference and damage. A 3 m length of tube was used to vent the captured methane away from the methane sampling point to avoid recirculation of methane gases. The methane analyzer was calibrated as per the manufacturer’s guideline at weekly intervals. Briefly, this calibration involved flushing of the unit with nitrogen gas to determine a true reading of 0% methane. Following this procedure standardized methane gas canisters at concentrations of 1%, 0.5% and 0.25% were used to calibrate and validate accurate measures were recorded by the instrument. 

Methane concentrations were recorded via a data logger (AEMC instruments, 4–20 m ADC, simple logger model SL20). The data logger was configured with 1 s measurement intervals.

### 2.4. Milk Quantity and Quality Measurements

This study was conducted on a commercial dairy farm with a robotic milking system installed which was capable of recording individual animal milk production data during the trial. Milk composition for individual cows was determined by the robot via the MQC sensors installed in the robot that generate an indication for protein and fat. This included daily milking history (average milk/day), milk composition data (fat indication, protein indication), and Bulk Milk Somatic Cell Count (BMCC), which was monitored via the routine analysis of the milk bulk tank at each collection.

#### 2.4.1. Animal Ethics Code Number

All the procedures involved in this study involved the treatment of animals, including handling. Polygain™ feeding and data collection were approved by the La Trobe University Ethics Committee (Approval No. AEC20028)

#### 2.4.2. Statistical Analysis Details

The results were analyzed using IBM SPSS Statistics software Version 28.0, applying general linear model with milk yield, milk composition and methane concentrations as dependent variables and baseline period and intervention periods as fixed factors. Statistical significance was set at *p* < 0.05.

## 3. Results

### 3.1. Milk Yield

**Figure 1:**
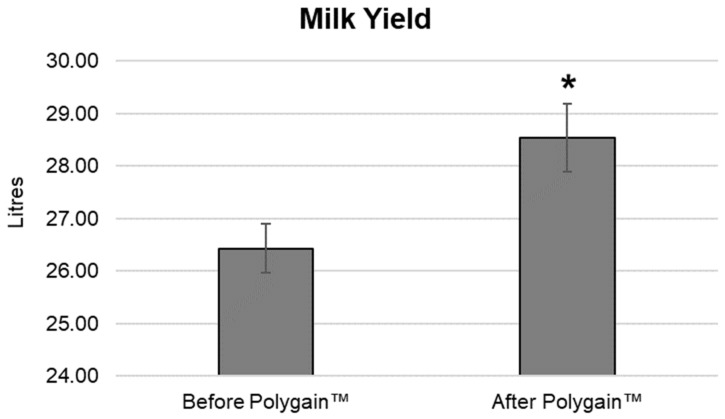
Weekly milk yield during the baseline collection phase (week 1–3, Before Polygain™) and during the following 3-week intervention period (week 4–6, After Polygain™). * indicates statistical significance (*p* < 0.05).

Milk yield during the baseline collection period was observed to average 26.43 litres, per cow, per day (Figure 1). This increased significantly in the intervention phase of the trial to 28.54 litres per cow per day (*p* < 0.05). This increase represents an increased milk yield of 7.4% on average per cow per day during the intervention phase. 

### 3.2. Methane Emissions

**Figure 2:**
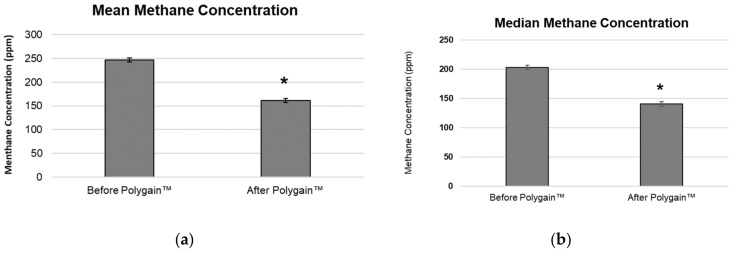
(**a**) Mean daily methane concentration during the baseline collection phase (week 1–3, before Polygain™) and during the following 3-week intervention period (week 4–6, After Polygain™). (**b**) Median daily methane concentration during the baseline collection phase (week 1–3, before Polygain™) and during the following 3iweek intervention period (week 4–6, After Polygain™). * Indicates statistical significance (*p* < 0.05).

The methane concentration detected in the baseline phase of the trial was a mean of 246.59 ppm (±4.15 SEM) (Figure 2a) and a median concentration of 202.96 ppm (±3.70 SEM) (Figure 2b). This concentration reduced significantly in the intervention phase of the trial to a methane concentration of 161.09 ppm (±4.34 SEM) and a median concentration of 140.51 (±3.86 SEM) (Figure 2a,b). This reduction represents a decreased methane concentration detected during the intervention phase of the trial of 30.8% change in the median methane concentration and 34.7% in the mean methane concentration.

### 3.3. Milk Composition

**Figure 3:**
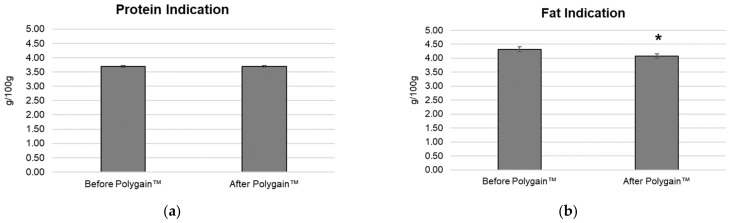
(**a**) Mean protein concentration during the baseline collection phase (week 1–3, before Polygain™) and during the following 3-week intervention period (week 4–6, After Polygain™). (**b**) Mean fat indication concentration during the baseline collection phase (week 1–3, before Polygain™) and during the following 3-week intervention period (week 4–6, After Polygain™). * Indicates statistical significance (*p* < 0.05).

There was no significant difference in protein content for the baseline and intervention phases of the trial (Figure 3a). The baseline protein indication was 3.69 g/100g compared to 3.69 g/100 g in the intervention phase of the trial. Fat indication decreased from 4.32 g/100 g to 4.03 g/100 g. This was a statistically significant difference of 6.7% (Figure 3b). 

**Figure 4:**
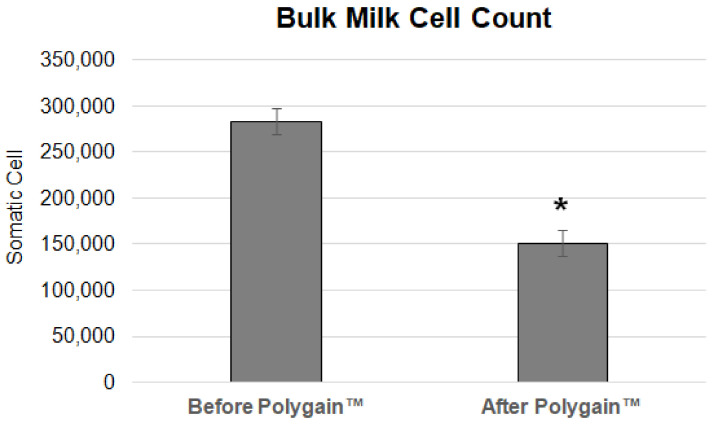
Bulk tank average somatic cell count for the 3-week intervention phase with the sugarcane extract (before polygain) compared to the average of the 3-week Polygain™ intervention. * Indicates statistical significance (*p* < 0.05).

Bulk milk cell count averaged 283,200 BMCC during the baseline phase (Figure 4). Following the intervention phase, the BMCC was detected to be at the significantly reduced level of 151,100 BMCC. 

## 4. Discussion

The present study indicated that the dietary inclusion of Polygain™ significantly increased the average milk yield, whilst significantly decreasing both methane concentration and bulk somatic cell counts. These results are particularly relevant to farm management practices as they have been demonstrated in a commercial dairy farm environment. 

This is the first study relating to the use of the novel feed ingredient, Polygain™, in dairy cattle. However, previous studies in other ruminant species have suggested comparable results to what is reported in the present study. A previous study, in second cross lambs, that also used a feeding rate of Polygain™ at 0.25% of DMI reported a methane reduction of 49%, compared to the 34.7% reduction reported in the present study [18]. It is notable that the reduced methane associated with increased production has now been observed in two distinct species of ruminant animals. The relatively low dosage of 0.25% of dry matter intake is an interesting finding in both species. This may enable the adoption of technology with minimal alterations to the typical diet and management practices on farms. These results demonstrate a comparable decrease in methane concentration detected in the feed bin during milking; however, it is worth evaluating the differences in these studies whilst comparing these results. There are several possible explanations for the difference in reduction observed in this study. One clear difference between these two studies is one study was conducted in a controlled research setting, and the other conducted a trial under commercial farm conditions. The measurement of methane under commercial settings remains a challenging task. There have been multiple approaches to the on-farm measurement of agricultural methane, with the strengths and weaknesses of each approach discussed in detail previously [20,22,23]. One of the major challenges for the measurement methodology used in the present study is that methane measurements are only completed while the animal is being milked. However, this technique enables an estimate of methane emission to be completed in commercial dairies. This is in contrast to other measurement techniques that are more applicable to research settings such as SF6 and respiration chambers that cannot be readily deployed into a commercial farm at scale. Additional work to verify these on-farm results using measurement techniques such as SF6 and respiration chambers would be a logical extension of this work. 

The hypothesized mechanism of action for the sugarcane extract, Polygain™, is related to the observed capability to fine tune the rumen microbiome. This fine tuning is hypothesized to favor the proliferation of microbes capable of converting energy in the diet of the animal to products such as milk and meat, whilst inhibiting the proliferation of methanogenic organisms such as the methanogenic archaea. This mechanism has been described in further detail, as reported previously [18]. In the present study, a significant increase in production was observed, which would potentially support the hypothesized mechanism of action. However, this does not offer direct evidence that a shift in rumen microbiome has occurred. The collection of rumen fluid and analysis would be required to accurately test this assumption. Due to the constraints of operating within a working dairy farm, it was not feasible to collect rumen fluid and assess whether the same underlying mechanisms were likely responsible for the milk yield and methane concentration observations made in the present study. Further studies conducted under controlled research conditions where this mechanism of action can be evaluated are recommended to follow up on this proposed mechanism of action. 

The present study is the first report of a significant reduction in bulk somatic cell count correlating with the dietary inclusion of Polygain™. Bulk tank somatic cell counts were taken every 3 days in this study. There may have been other contributing factors to the bulk tank somatic cell count reduction beyond the introduction of Polygain™. However, the direct correlation with the introduction of Polygain™ and this rapid decline is worthy of further examination. One of the limitations of this on farm study, is that no individual animal cell count data were available, only the bulk tank. Further investigations into this potential preventative solution for mastitis at an individual animal level would be an advisable follow-up study. In addition to the potential benefits to animal health and welfare of reducing somatic cell count, there is a potential benefit to milk stability and shelf life from reducing the somatic cell count in milk. Previous studies have observed a decreased stability of milk products with an increasing somatic cell count in raw milk [24]. Therefore, any follow-up studies could benefit from assessing any effect on milk stability and milk quality that may be associated with this observed reduction in somatic cell count. 

Polyphenols have previously been described as potential solutions to prevent or even treat mastitis [25,26,27,28,29]. However, the majority of this work has been under in vitro settings, in non-ruminant mammalian models such as mice or required intramammary delivery. It is very surprising that a feed additive delivered orally could be correlated with such a significant effect on bulk tank somatic cell counts. The present study observation of a rapid and highly significant decrease in bulk somatic cell count under commercial conditions is a major finding that warrants further investigation. The opportunity to use a non-antibiotic solution to combat mastitis presents an exciting opportunity for the dairy industry. Consumers are increasingly demanding information relating to the principles of antibiotic stewardship used to produce food products. A natural solution for promoting improved udder health, with reduced reliance on antibiotics, would be of significant benefit to producers. 

## 5. Conclusions

The inclusion of Polygain™ to the diets of dairy cattle at a relatively low dosage of 0.25% of total dry matter intake increased milk production significantly, whilst decreasing methane and bulk somatic cell counts. These observations were under commercial on-farm conditions enabling a direct path for adoption by dairy farmers. Further work is required to understand the effect of the Polygain™ product under different dietary conditions, animal genetics and seasonal conditions.

## Data Availability

Data available on request.
